# A new family of “megaphages” abundant in the marine environment

**DOI:** 10.1038/s43705-021-00064-6

**Published:** 2021-10-20

**Authors:** Slawomir Michniewski, Branko Rihtman, Ryan Cook, Michael A. Jones, William H. Wilson, David J. Scanlan, Andrew Millard

**Affiliations:** 1 Warwick Medical School, University of Warwick, Gibbet Hill Road, Coventry, CV4 7AL UK; 2 School of Life Sciences, University of Warwick, Gibbet Hill Road, Coventry, CV4 7AL UK; 3 School of Veterinary Medicine and Science, University of Nottingham, Sutton Bonington Campus, Sutton Bonington, Leicestershire LE12 5RD UK; 4 Marine Biological Association, The Laboratory, Plymouth, United MBA, Plymouth, UK; 5 School of Biological and Marine Sciences, University of Plymouth, Plymouth, PL4 8AA UK; 6 Dept Genetics and Genome Biology, University of Leicester, University Road, Leicester, LE1 7RH UK

**Keywords:** Environmental sciences, Bacteriophages, Water microbiology

## Abstract

Megaphages, bacteriophages harbouring extremely large genomes, have recently been found to be ubiquitous, being described from a variety of microbiomes ranging from the animal gut to soil and freshwater systems. However, no complete marine megaphage has been identified to date. Here, using both short and long read sequencing, we assembled >900 high-quality draft viral genomes from water in the English Channel. One of these genomes included a novel megaphage, Mar_Mega_1 at >650 Kb, making it one of the largest phage genomes assembled to date. Utilising phylogenetic and network approaches, we found this phage represents a new family of megaphages. Genomic analysis showed Mar_Mega_1 shares relatively few homologues with its closest relatives, but, as with other megaphages Mar_Mega_1 contained a variety of auxiliary metabolic genes responsible for carbon metabolism and nucleotide biosynthesis, including a NADP-dependent isocitrate dehydrogenase [Idh] and nicotinamide-nucleotide amidohydrolase [PncC], which have not previously been identified in megaphages. Mar_Mega_1 was abundant in a marine virome sample and related phages are widely prevalent in the oceans.

## Introduction

Phages, viruses that prey on bacteria, are the most abundant biological entities on Earth. Although they are ubiquitous and highly diverse components of the microbiome [1], the majority of known phages contain genomes smaller than 200 kb [2]. However, advances in the field of viral metagenomics led to the recent discovery of megaphages—phages with extremely large genomes (>540 kb in length). The first identified megaphages, Lak phages, contained alternatively coded genomes and were present in both animal and human gut microbiota [3]. This was followed by the identification of multiple megaphage genomes including the largest known phage genome, 735 kb in length, from a range of viromes across Earth’s ecosystems [4]. Currently megaphages have been found in human and animal microbiomes [3, 5] soil and deep subsurface environments [4] and freshwater lakes [4, 6]. However, megaphages thus far have not been described from marine systems.

## Results

**Fig. 1:**
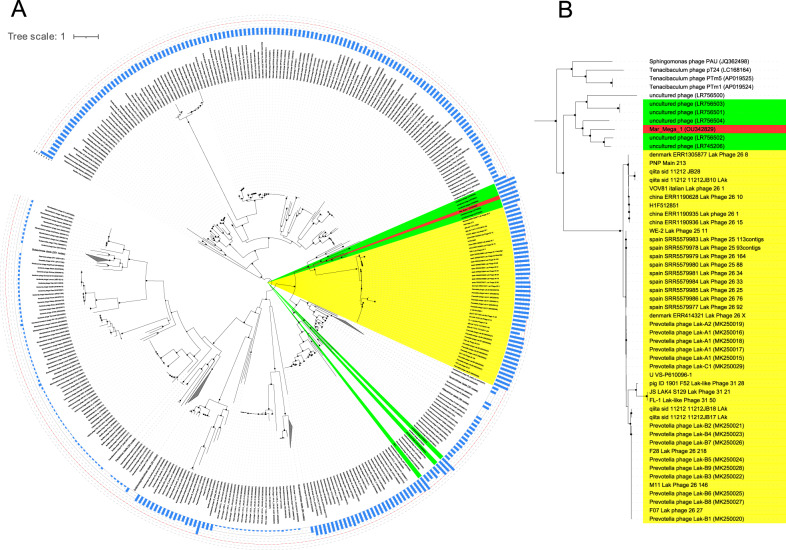
Phylogeny of megaphages. **A** Phylogenetic analysis of selected phages and  all megaphages (including incomplete megaphage genomes) based on the amino acid sequence of the terminase large subunit. Protein sequences were aligned with MAFFT and trees constructed with IQ-TREE, based on the LG + R7 model of evolution with 1000 bootstrap replicates. Mar_Mega_1 is marked in red, Lak phages in yellow and other megaphages in green. Filled circles denote bootstrap values >70%, with the size of the circle proportional to the bootstrap value. Some clades have been collapsed for clarity. **B** Subtree containing Mar_Mega_1.

Using a combination of Illumina and MinION sequencing, three marine viral communities isolated from the western English Channel and Plymouth Sound were investigated (Supplementary Methods). This resulted in the reconstruction of 23,179 putative viral contigs (acc:ERZ2485795) with 972 high-quality draft genomes of which 367 are predicted to be complete based on MIUViG standards [7] (Table S1). These included six phage genomes with lengths > 200 kb and one exceptionally large 656,628 bp genome, Mar_Mega_1 (acc:OU342829.1). This genome comprised 1062 predicted genes including one tmRNA, 50 tRNAs and 1011 coding sequences. However, a combination of approaches including Blast, hmm searching and Phyre2 analyses resulted in the function being assigned to only 268 proteins (Table S2). The large genome size of Mar_Mega_1 makes it one of the largest phage genomes assembled to date and puts it in the range of “megaphages”. Comparison of the Mar_Mega_1 genomic sequence with known phages showed no significant similarity at the nucleotide level. Clustering with other phages using vContact2 suggested it is related to other megaphages (Fig. S1, Table S3). This was confirmed by a phylogeny built using the amino-acid sequence of the terminase large subunit (Fig. 1). All Lak-like megaphages formed a single clade, as was previously described [3], whilst Mar_Mega_1 formed a cluster with the largest megaphages (genome lengths > 630 kb). Our newly identified phage is in a sister group to the uncultured phages LR756502 and LR745206 [4] identified from a freshwater lake in France and sub-surface sample in Japan, respectively. However, the long branch lengths suggest that Mar_Mega_1 is only distantly related to these freshwater megaphages.

To further investigate the relationship of Mar_Mega_1 with its closest relatives, a core gene approach was used using parameters recommended for defining phage families [8]. All three phages share a core of only 125 genes that constitutes between 12.3% (Mar_Mega_1) and 13.2% (LR745206) of genes in each phage suggesting these phages do not form a single family based on current definitions [8]. Phages LR756502 and LR745206 share nearly twice as many genes with each other (30.6–31.5%) than either phage does with Mar_Mega_1 (14.4–17.5%). This suggests Mar_Mega_1 represents a new family of phages in the megaphage size range based on current standards [8] (Fig. S2).

**Fig. 2:**
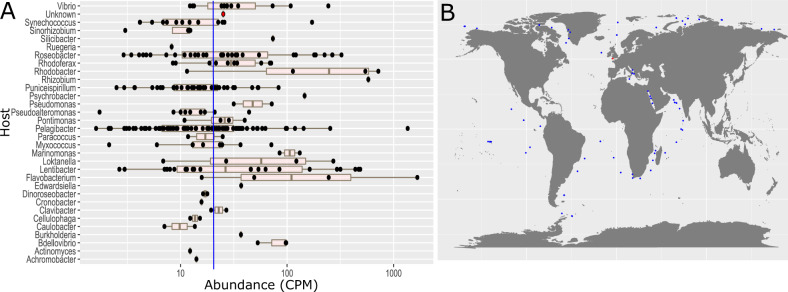
Abundance and distribution of Mar_Mega_1 -like phages. **A** Relative abundance of vOTUs associated with known phages in the Plymouth Sound virome. Abundance is represented by counts per million. Each black dot represents a viral contig that could be associated with a known phage based on mash similarity, or clusters in the same group as a known phage using vContact2. Host information was extracted from known phages. Mar_Mega_1 is represented by a red dot (unknown host). The median relative abundance of all 269 vOTUs with similarity to known phages is denoted by a blue horizontal line. **B** The distribution of Mar_Mega_1-like phages. Reads from GOS and GOV2.0 were mapped against Mar_Mega_1 and 9 TARA vOTUs carrying a TerL that clustered with Mar_Mega_1. Stations are marked where read coverage was >1x across 70% of the genome. Blue dots denote where Mar_Mega_1-like phages were detected and red where Mar_Mega_1 was detected.

Having established Mar_Mega_1 as the first representative of a new family, we sought to establish its distribution in the marine environment. Although Mar_Mega_1 was present only in the samples taken from Plymouth Sound (Table S1), we have estimated that it is as abundant as cultivated phages that infect marine bacteria such as *Pelagibacter* and *Synechococcus* (e.g. *Synechococcus* phage S-SKS1*, Pelagibacter* phage HTVC115P and Lentibacter phage vB_LenP_ICBM2) through read mapping (Fig. 2). However, the abundance of Mar_Mega_1 might be underestimated as the majority of virions larger than 0.22 µm should have been removed during the filtration step. As no megphages have been cultured to date their virion size remains unknown, but it is probable they have larger capsids. Using the TerL sequence from Mar_Mega_1 to query the TARA contigs via BlastP, phylogenetic analysis revealed a further nine phages that are sister to the group containing Mar_Mega_1, suggesting related phages are present in the TARA oceans dataset [9] (Fig. S3A, B, Table S4). As these proteins were found on genome fragments, it was not possible to compare total genome content. However, their placement within the same cluster suggests closely related phages are present in the marine environment. Moreover, CheckV analysis of the genome fragments supports the hypothesis they are fragments of much larger phages (Table S4). Furthermore, using contigs on which the TerL homologues were identified with a read mapping approach the prevalence of Mar_Mega_1-like phages in TARA and GOV2.0 viromes was investigated (Fig. 2B, Table S5) [9, 10]. Despite collectively several thousands of reads mapping to Mar_Mega_1, no single sample passed the accepted threshold of >1x coverage across 70% of the genome [11]. In contrast, we found contigs carrying TerL homologues to be widely distributed across the 162 marine stations (Fig. 2B, Table S5). Thus, whilst a marine origin of this megaphage family is likely, because of the abundance of Mar_Mega_1 only in our Plymouth Sound sample we cannot rule out a freshwater source for this phage given the close proximity of this site to a river estuary.

Several methods were tested to determine a putative host for Mar_Mega_1 (see Supplementary Methods). However, no host could be predicted with a high degree of certainty. Genomic analysis of Mar_Mega_1 identified several proteins detected in other megaphages including phage structural proteins and phage replication proteins (Table S2). Unlike some previously identified megaphages no CRISPR-cas system was identified [3]. However, a range of auxiliary metabolic genes (AMGs) were detected, homologues of which have not yet been identified in other megaphages (Table S2). These included putative nicotinamide-nucleotide amidohydrolase [PncC], NADP-dependent isocitrate dehydrogenase [Idh] and patatin-like phospholipase (PLP) [12] enzymes. In addition, Mar_Mega_1 possessed a putative TonB-dependent receptor (SusC) which was also present in LR745206, as well as AMGs encoding putative dihydrofolate reductase, phosphoesterase and peptidase enzymes which were also found in megaphages LR745206, LR756501, LR756502, LR756503 and LR756504 (Table S2).

The presence of AMGs potentially involved in carbon metabolism in Mar_Mega_1 is consistent with previous research indicating the prevalence of AMGs responsible for carbohydrate and amino acid uptake and metabolism in model marine phage systems and viral metagenomes [13]. For example, the TonB-dependent receptor SusC might be responsible for increasing carbohydrate uptake during infection [14], whereas the NADP-dependent isocitrate dehydrogenase [Idh], an AMG which was previously detected in marine viromes [15] carries out the oxidative decarboxylation of isocitrate to α-ketoglutarate (αKG). αKG is a rate-determining intermediate in the tricarboxylic acid cycle and crucial for both cellular energy metabolism and as a source of glutamate and glutamine. As such, it is a central regulator affecting numerous metabolic pathways through its role in bridging carbon and nitrogen metabolism, as well as being a key signalling molecule of cellular nutrient status [16]. Thus this enzyme potentially plays an important role during the infection process. Furthermore, AMGs responsible for pyridine nucleotide synthesis such as nicotinamide-nucleotide amidohydrolase (PncC) whilst new to megaphages, have previously been found in other phages such as *Vibrio* phage KVP40 which encodes its own NAD^+^ salvage pathway [17]. Moreover, the Mar_Mega_1 phage encoded dihydrofolate reductase could act two-fold by increasing the host’s capacity to convert dihydrofolate into tetrahydrofolate which is essential for purine nucleotide biosynthesis or, due to similarity with a putative *dfrA3* antibiotic resistance gene, confer protection against diaminopyrimidine antibiotics, which are one of the most common antibiotic pollutants in marine environments [18].

This is the first time a patatin-like phospholipase (PLP) was identified within a phage genome. Although the function of PLPs is currently not clear, a role in bacterial pathogen–eukaryotic host interactions was suggested [19]. We have since been able to identify a homologue of PLP in other phages (acc: LR745206), suggesting that megaphages might increase the virulence of their putative bacterial hosts.

## Conclusions

We identified the largest marine megaphage to date. Using phylogenetic and genomic analyses it is distantly related to megaphages found in other environments. Analysis of marine viromes suggests Mar_Mega_1-like phages are abundant and widely distributed in the marine environment.

## Supplementary information

Mar_Mega_1like contigs

Supplementary Methods

Supplementary Figure Legends

Figure S1

Figure S2

Figure S3A

Figure S3B

Table S1

Table S2

Table S3

Table S4

Table S5
