# Supplementary material for: A new family of “megaphages” abundant in the marine environment
Source: ISME Commun. 2021 Oct 20;1:58. doi: 10.1038/s43705-021-00064-6 (PMC9723777; doi:10.1038/s43705-021-00064-6)
Supplement: Supplementary file 2 — Supplementary Methods [file 43705_2021_64_MOESM2_ESM.docx]

**Supplementary Information**

**Samples: collection and DNA extraction**

Three marine water samples were collected (16.04.2019): Western Channel Observatory station L4 (50°15'00.0"N 4°13'01.2"W) surface waters, station L4 at a depth of 25 metres and Plymouth Sound (50°21'44.4"N 4°09'55.2"W) surface waters. Water samples were filtered using 0.22 µm pore-size cellulose acetate membrane filters (Corning) to remove bacterial cells and larger particles. However, since megaphages have not been cultured their virion size is unknown. Thus, their large genome size may result in larger capsids being excluded by the 0.22 µm pore-size filtration step. Subsequently, Amicon Ultra-15 100K (100,000 MWCO) concentrator columns (Millipore) were used to concentrate 4.5 L of each water sample, followed by 30 mL of SM wash buffer to remove the excess of marine salts. The resulting 1 mL of permeate containing concentrated and cleaned up marine viral fraction in SM buffer was stored at 4°C. Non-encapsidated nucleic acids were removed by mixing 900 µL of each sample with 100 µL 10x DNase buffer and supplemented with 2 µL (4 U) of DNase I (NEB) and 1 U of RNase, followed by 30 min incubation at 37°C. Phage DNA was extracted using phenol:chloroform:isoamyl alcohol [[1]](https://paperpile.com/c/dC1otI/ZJgs), and stored at -20°C for further analysis.

**Short read sequencing (Illumina HiSeq)**

Short read sequencing of the DNA samples was performed by the University of Liverpool Centre for Genomic Research using the Illumina HiSeq4000 platform (2 x 150 bp). Subsequently, Cutadapt v.1.2.1 (Martin, 2011) with `-O 3` option was used to remove Illumina adapters from the resulting Fastq, followed by trimming using Sickle v.1.200 [[2]](https://paperpile.com/c/dC1otI/WGk5) with default options. Metagenomic contigs were assembled de novo using a previously described pipeline (Roux *et al.*, 2019) as follows: tadpole.sh v.37.54 [[3]](https://paperpile.com/c/dC1otI/PjWa) with `mode=correct ecc=t prefilter=2` was used for read correction followed by clumpify.sh v.37.54 [[3]](https://paperpile.com/c/dC1otI/PjWa) using `dedupe subs=0 passes=2` options to remove identical reads. Finally, concatenated reads from all three samples were cross-assembled using SPAdes v.3.13.0 [[4]](https://paperpile.com/c/dC1otI/v7S9) with `--sc --only-assembler -k 77,99,127` options.

**Long read sequencing (MinION)**

Due to the low amount of available DNA and the high requirement for long-read sequencing an aliquot of each DNA sample underwent whole genome amplification with the Illustra Ready-To-Go GenomiPhi V3 DNA Amplification Kit (GE Healthcare) following the manufacturer’s protocol. S1 Nuclease (Thermo Fisher Scientific) digestion was subsequently performed to de-branch chimeric DNA formed during the rolling circle amplification process ([[5]](https://paperpile.com/c/dC1otI/vqu2)). Resulting dsDNA was cleaned up using a DNA Clean & Concentrator Kit (Zymo Research) and used as an input for MinION sequencing.

MinION libraries were prepared using EXP-NBD104 and SQK-LSK109 kits (Oxford Nanopore) following the manufacturer’s protocol for Native barcoding genomic DNA. Libraries were sequenced using a MinION with a FLO-MIN106 flowcell and MinIT (Oxford Nanopore) for data storage and analysis. Bases were called with guppy_basecaller v.3.0.3 software using dna_r9.4.1_450bps_hac.cfg configuration for high accuracy calling, followed by demultiplexing of the samples with guppy_barcoder v.3.0.3 using default configuration and trimming of the barcodes with Porechop v.0.2.1 [[6]](https://paperpile.com/c/dC1otI/C7eH). Genome assembly was performed using Flye v.2.6 [[7]](https://paperpile.com/c/dC1otI/Ch0C) using `--nano-raw --meta --genome-size 5g --t 90` options. Subsequently, minimap2 v.2.14 [[8]](https://paperpile.com/c/dC1otI/UtOB) with `-ax map-ont` options was used to map MinION reads to the assembly.fasta file, followed by polishing of the output with marginPolish v.1.0.0 (Paten and Pesout, 2019) using `allParams.np.ecoli.json` option. Finally, the resulting assembly was further polished with Illumina short reads using Pilon v.1.23 (Walker *et al.*, 2014) using default options.

**Analysis of assembled metagenomes**

Both Illumina and MinION metagenomics assemblies were concatenated, and duplicate sequences and sub-sequences at >95% identity cut-off were removed with dedupe.sh [[3]](https://paperpile.com/c/dC1otI/PjWa) using the `minidentity=95` option. This was followed by the removal of contigs shorter than 5,000 bp, which are unlikely to represent complete phage sequences. Remaining contigs were scanned with DeepVirFinder v.1.0 [[9]](https://paperpile.com/c/dC1otI/TvWp) using default options with a p value cut-off of <0.05 for the presence of phage sequences, followed by the checkV v.0.6 [[10]](https://paperpile.com/c/dC1otI/hQqg) analysis to determine the quality of the reconstructed phage genomes using: contamination, completeness, repeats and quality_summary commands with default options.

Reconstructed megaphage gene sequences were predicted and annotated with Prokka v.1.11 [[11]](https://paperpile.com/c/dC1otI/nCzz) using a custom database of all phage genomes [[12]](https://paperpile.com/c/dC1otI/Y8IV) that had previously been extracted from GenBank (Jul 2017) [[12]](https://paperpile.com/c/dC1otI/Y8IV). The resulting protein sequences were subsequently combined with protein sequences of all known phages and megaphages (Table S3) at the time of the analysis (January 2021) and used as an input for vContact2 v.0.9.15 [[13]](https://paperpile.com/c/dC1otI/b24A) analysis with ‘--rel-mode Diamond --db ProkaryoticViralRefSeq94-Merged --pcs-mode MCL --vcs-mode ClusterONE’ options. A network map was visualised using Cytoscape v.3.7.2 [[14]](https://paperpile.com/c/dC1otI/nMpK). Phylogenetic analysis of selected phages and all megaphages was based on the amino-acid sequence of the terminase large subunit. Protein sequences were aligned using MAFFT v7.271 [[15]](https://paperpile.com/c/dC1otI/fhjG) with default options followed by phylogenetic tree construction using IQ-TREE v.1.6.3 [[16]](https://paperpile.com/c/dC1otI/hf03) using the `--bb 1000` option, with models of evolution selected using modeltest [[17]](https://paperpile.com/c/dC1otI/QKnH). The resulting tree was visualised using ITOL [[18]](https://paperpile.com/c/dC1otI/peR9). ANI was determined using the Kostas lab online ANI calculator [[19]](https://paperpile.com/c/dC1otI/ZjJs). The closest known relatives vOTUs were identified by taking the top hit when compared to a MASH database of current phage genomes [[12]](https://paperpile.com/c/dC1otI/Y8IV). The following mash settings were used: `mash -dist -d 0.3 -s 1000 -p 90` [[20]](https://paperpile.com/c/dC1otI/T6C5). Putative hosts of all vOTUs were predicted by comparison of vOTU contigs to a database of known CRISPR-spacers, allowing up to 3 mismatches in spacers [[21]](https://paperpile.com/c/dC1otI/pFyt) .

Manual annotation of genes in Mar_mega_1 was carried out using Phyre2 [[22]](https://paperpile.com/c/dC1otI/2wYt) and HHpred [[23]](https://paperpile.com/c/dC1otI/lTLj) to predict the function of the large number of hypothetical proteins. Genomic comparison of Mar_Mega_1 with its two closest uncultured relatives (accession LR756502 and LR756502) [[24]](https://paperpile.com/c/dC1otI/CcEj) was carried out using get_homologues, with the following settings -M -t 0 -A [[25]](https://paperpile.com/c/dC1otI/anXe).

### **Identification of TerL homolouges in TARA dataset**

Homologues of Mar_Mega_1 TerL were searched for in the TARA oceans dataset. Previously it has been shown the re-assembly of the TARA dataset can improve identification of viral contigs [26]. Therefore, we reassembled the TARA dataset using the parameters suggested [27] .Briefly adapters were trimmed with bbduk.sh `mink=11 tbo tpe k=23 hdist=1 hdist2=1 ftm=5` , followed by quality triming with bbduk.sh `maq=8 maxns=1 minlen=40 minlenfraction=0.6 k=27 hdist=1 trimq=12 qtrim=rl` , correction of errors with tadpole.sh `mode=correct ecc=t prefilter=2`.Individual libraries were assembled with megahit `megahit -k-min 21 --k-max 121 --k-step 20 -t 90 -m 0.9`. Assemblies were combined and identical contigs removed with depupe.sh. Contigs were filtered for a minimum length of 1 kb . Genes were called on each contig with prokka `--meta --noannotation`. A blastp database was created with makeblastdb using default settings. Blastp was carried out using the TerL from Mar_mega_1 as a query with a minimum evalue of 0.0001.

**Viral abundance analysis**

Viral abundance in samples collected in this study was determined by read mapping. Only short reads were used for the estimation of abundance to avoid bias introduced by amplification required for long read sequencing. Reads were mapped with bbmap.sh within minid=95 [[3]](https://paperpile.com/c/dC1otI/PjWa). Viral abundance was determined using CPM (counts per million) as follows. For each contig with coverage >=70% and average fold >1.0x its number of reads was divided by the contigs length in kb resulting in a reads per kilobase (RPK) value. CPM values were subsequently determined by dividing the RPK of each contig by the sum of all RPK values in the sample divided by a million. This value represented the abundance of each reconstructed phage genome normalised to the genome length and the total amount of reads obtained for each sample.

All raw data and supplementary data can be accessed via the ENA project number PRJEB34741 and Figshare at DOI: 10.25392/leicester.data.15022284

**Presence of Mar_mega1-like phages in TARA ocean and GOV2.0 datasets**

Following the identification of nine TerL homologues, we extracted the contigs (vOTUs) the genes were found upon. The presence of vOTUs in each TARA/GOV2.0 sample was assessed by read coverage, using a minimum of 1x coverage across 70% of the genome as commonly used in other studies (27). Reads from each TARA/GOV2.0 samples were downloaded from the SRA and mapped with bbmap.sh (Table S3).

### **Phage Families**

A pathway for bacteriophage classification has recently been described (Turner et al. 2021). Following these guidelines a new genera is defined as >70% ANI over 100% of the genome. As these phages do not meet this criteria, they will sit at a taxonomic level above this. Family level groups must also be monophyletic. Furthermore, they must share a significant gene content with other members of the family. The two phage LR756502 and LR745206 share ~30% of their genes, comparable with the percentage of genes in other recently proposed phage families (https://talk.ictvonline.org/ ). In contrast, Mar_mega1 shares only approximately half this amount with its closest relative. Given the lack of significant gene sharing Mar_mega1 likely represents a new family based on current criteria. This was further confirmed using VICTOR analysis that uses whole genome distances to identify families, with d_4_ settings for genome [28]*.* (Figure S2)

1. [Cook R, Hooton S, Trivedi U, King L, Dodd CER, Hobman JL, et al. Hybrid assembly of an agricultural slurry virome reveals a diverse and stable community with the potential to alter the metabolism and virulence of veterinary pathogens. *Microbiome* 2021; **9**: 65.](http://paperpile.com/b/dC1otI/ZJgs)

2. [Joshi NA, Fass JN, Others. Sickle: A sliding-window, adaptive, quality-based trimming tool for FastQ files (Version 1.33)[Software]. 2011.](http://paperpile.com/b/dC1otI/WGk5)

3. [Bushnell B. BBMap: A fast, accurate, splice-aware aligner. 2014. Lawrence Berkeley National Lab. (LBNL), Berkeley, CA (United States).](http://paperpile.com/b/dC1otI/PjWa)

4. [Bankevich A, Nurk S, Antipov D, Gurevich A a., Dvorkin M, Kulikov AS, et al. SPAdes: A new genome assembly algorithm and its applications to single-cell sequencing. *J Comput Biol* 2012; **19**: 455–477.](http://paperpile.com/b/dC1otI/v7S9)

5. [Lasken RS, Stockwell TB. Mechanism of chimera formation during the Multiple Displacement Amplification reaction. *BMC Biotechnol* 2007; **7**: 1–11.](http://paperpile.com/b/dC1otI/vqu2)

6. [Wick RR. Porechop.​ Github https://github com/rrwick. 2017. Porechop.](http://paperpile.com/b/dC1otI/C7eH)

7. [Kolmogorov M, Bickhart DM, Behsaz B, Gurevich A, Rayko M, Shin SB, et al. metaFlye: scalable long-read metagenome assembly using repeat graphs. *Nat Methods* 2020; **17**: 1103–1110.](http://paperpile.com/b/dC1otI/Ch0C)

8. [Li H. Minimap2: pairwise alignment for nucleotide sequences. *Bioinformatics* 2018; **34**: 3094–3100.](http://paperpile.com/b/dC1otI/UtOB)

9. [Ren J, Song K, Deng C, Ahlgren NA, Fuhrman JA, Li Y, et al. Identifying viruses from metagenomic data by deep learning. 2018.](http://paperpile.com/b/dC1otI/TvWp) arXiv:1806.07810

10. [Nayfach S, Camargo AP, Schulz F, Eloe-Fadrosh E, Roux S, Kyrpides NC. CheckV assesses the quality and completeness of metagenome-assembled viral genomes. *Nat Biotechnol* 2021; **39**: 578–585.](http://paperpile.com/b/dC1otI/hQqg)

11. [Seemann T. Prokka: Rapid prokaryotic genome annotation. *Bioinformatics* 2014; **30**: 2068–2069.](http://paperpile.com/b/dC1otI/nCzz)

12. [Cook R, Brown N, Redgwell T, Rihtman B, Barnes M, Clokie M, et al. INfrastructure for a PHAge REference Database: Identification of large-scale biases in the current collection of phage genomes. *bioRxiv* . 2021. , 2021.05.01.442102](http://paperpile.com/b/dC1otI/Y8IV)

13. [Bin Jang H, Bolduc B, Zablocki O, Kuhn JH, Roux S, Adriaenssens EM, et al. Taxonomic assignment of uncultivated prokaryotic virus genomes is enabled by gene-sharing networks. *Nat Biotechnol* 2019.](http://paperpile.com/b/dC1otI/b24A)

14. [Shannon P, Markiel A, Ozier O, Baliga NS, Wang JT, Ramage D, et al. Cytoscape: a software environment for integrated models of biomolecular interaction networks. *Genome Res* 2003; **13**: 2498–2504.](http://paperpile.com/b/dC1otI/nMpK)

15. [Katoh K, Standley DM. MAFFT multiple sequence alignment software version 7: improvements in performance and usability. *Mol Biol Evol* 2013; **30**: 772–780.](http://paperpile.com/b/dC1otI/fhjG)

16. [Nguyen L-T, Schmidt HA, von Haeseler A, Minh BQ. IQ-TREE: A fast and effective stochastic algorithm for estimating maximum-likelihood phylogenies. *Mol Biol Evol* 2015; **32**: 268–274.](http://paperpile.com/b/dC1otI/hf03)

17. [Posada D, Crandall KA. MODELTEST: testing the model of DNA substitution. *Bioinformatics* 1998; **14**: 817–818.](http://paperpile.com/b/dC1otI/QKnH)

18. [Letunic I, Bork P. Interactive Tree Of Life (iTOL): An online tool for phylogenetic tree display and annotation. *Bioinformatics* 2007; **23**: 127–128.](http://paperpile.com/b/dC1otI/peR9)

19. [Rodriguez-R LM, Konstantinidis KT. Bypassing cultivation to identify bacterial species. *Microbe Wash DC* 2014; **9**: 111–118.](http://paperpile.com/b/dC1otI/ZjJs)

20. [Ondov BD, Treangen TJ, Melsted P, Mallonee AB, Bergman NH, Koren S, et al. Mash: fast genome and metagenome distance estimation using MinHash. *Genome Biol* 2016; **17**: 132.](http://paperpile.com/b/dC1otI/T6C5)

21. [Dion MB, Plante P-L, Zufferey E, Shah SA, Corbeil J, Moineau S. Streamlining CRISPR spacer-based bacterial host predictions to decipher the viral dark matter. *Nucleic Acids Res* 2021; **49**: 3127–3138.](http://paperpile.com/b/dC1otI/pFyt)

22. [Kelley LA, Mezulis S, Yates CM, Wass MN, Sternberg MJE. The Phyre2 web portal for protein modeling, prediction and analysis. *Nat Protoc* 2015; **10**: 845–858.](http://paperpile.com/b/dC1otI/2wYt)

23. [Söding J, Biegert A, Lupas AN. The HHpred interactive server for protein homology detection and structure prediction. *Nucleic Acids Res* 2005; **33**: W244–8.](http://paperpile.com/b/dC1otI/lTLj)

24. [Devoto AE, Santini JM, Olm MR, Anantharaman K, Munk P, Tung J, et al. Megaphages infect *Prevotella* and variants are widespread in gut microbiomes. *Nature Microbiology* 2019.](http://paperpile.com/b/dC1otI/CcEj)

25. [Contreras-Moreira B, Vinuesa P. GET_HOMOLOGUES, a versatile software package for scalable and robust microbial pangenome analysis. *Appl Environ Microbiol* 2013; **79**: 7696–7701.](http://paperpile.com/b/dC1otI/anXe)

26. [Flores-Uribe J, Philosof A, Sharon I, Fridman S, Larom S, Béjà O. A novel uncultured marine cyanophage lineage with lysogenic potential linked to a putative marine *Synechococcus* ‘relic’ prophage. *Environ Microbiol Rep* 2019; **11**: 598–604.](http://paperpile.com/b/dC1otI/P8Ev)

27. [Roux, S., Trubl, G., Goudeau, D., Nath, N., Couradeau, E., Ahlgren, N. A., Zhan, Y., Marsan, D., Chen, F., Fuhrman, J. A., Northen, T. R., Sullivan, M. B., Rich, V. I., Malmstrom, R. R., & Eloe-Fadrosh, E. A. Optimizing de novo genome assembly from PCR-amplified metagenomes. *PeerJ* 2019; **7**.](http://paperpile.com/b/dC1otI/FUQe)

28. Meier-kolthof JP, Göker M. VICTOR : Genome-based Phylogeny and Classification of Prokaryotic Viruses. Bioinformatics 2017; 33: 3393–3404.
