# Supplementary material for: A new family of “megaphages” abundant in the marine environment
Source: ISME Commun. 2021 Oct 20;1:58. doi: 10.1038/s43705-021-00064-6 (PMC9723777; doi:10.1038/s43705-021-00064-6)
Supplement: Supplementary file 3 — Supplementary Figure Legends [file 43705_2021_64_MOESM3_ESM.docx]

**Supplementary Figure Legends**

Figure S1. Network map of Mar_Mega_1 in relation to known phages. Node colours denote reference (NCBI) phage genomes (teal), Lak phages (yellow), megaphages (green) and Mar_Mega_1 (red).

Figure S2. VICTOR analysis of megaphages identified to be related to Mar_Mega_1.

Figure S3. A) Phylogenetic analysis of selected phages and all megaphages (including incomplete megaphage genomes) and TerL homologues identified in the TARA dataset based on the amino acid sequence of the terminase large subunit. B) Zoomed in clade of Mar_Mega_1-like phages

TableS1. List of metagenomic contigs reconstructed in this study. The dataset includes contig names, lengths, CheckV quality and completeness analysis, abundances (CPM) and closest known phage relative determination (where applicable).

TableS2. Functional annotation of the Mar_Mega_1 genome using Prokka and Phyre2 approaches. The dataset includes BlastP analysis and homologue search results for selected putative auxiliary metabolic genes.

TableS3. List of megaphages and Lak phages (including incomplete megaphage genomes) used in vContact2 and phylogenetic analyses.

TableS4 List of reassembled TARA contigs containing megaphage-like TerL. The dataset includes contig length, predicted contig completeness, TARA reads sample ID and collection location metadata.

Table S5. Results of mapping GOV2 reads to the reassembled TARA contigs. The dataset contains the ID of the reassembled TARA contig, GOV2 reads sample ID, GOV2 sample collection location and mapping results.
