# Supplementary figures and images for: A new family of “megaphages” abundant in the marine environment
Source: ISME Commun. 2021 Oct 20;1:58. doi: 10.1038/s43705-021-00064-6 (PMC9723777; doi:10.1038/s43705-021-00064-6)

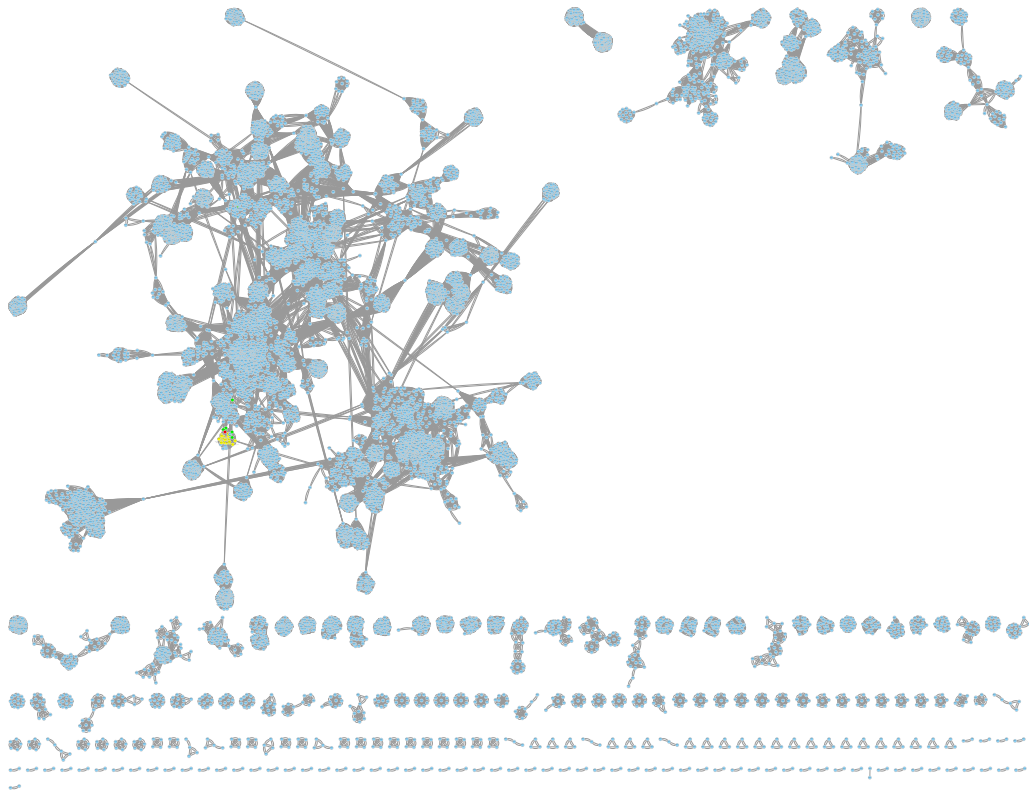

Supplement: Supplementary file 4 — Figure S1 [file 43705_2021_64_MOESM4_ESM.pdf]

Tree scale: 0.01

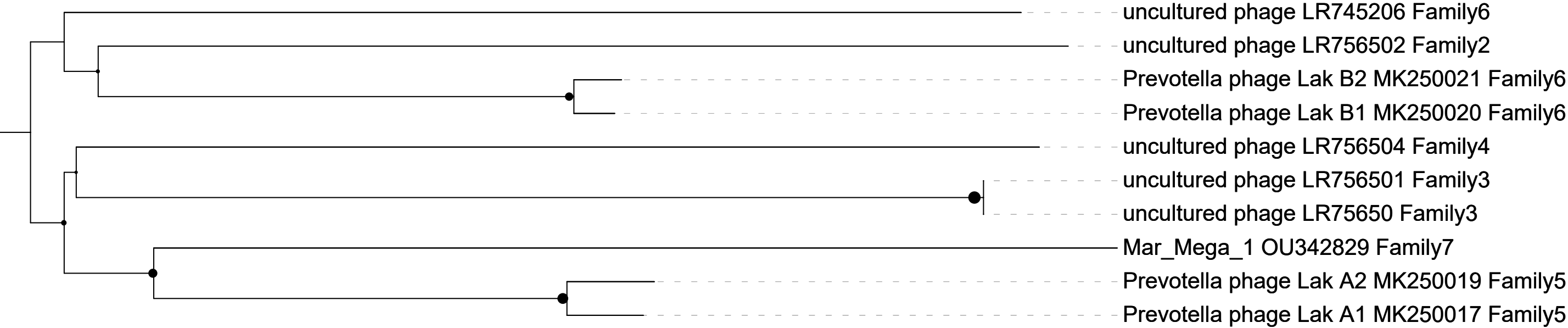

Supplement: Supplementary file 5 — Figure S2 [file 43705_2021_64_MOESM5_ESM.pdf]

Tree scale: 1

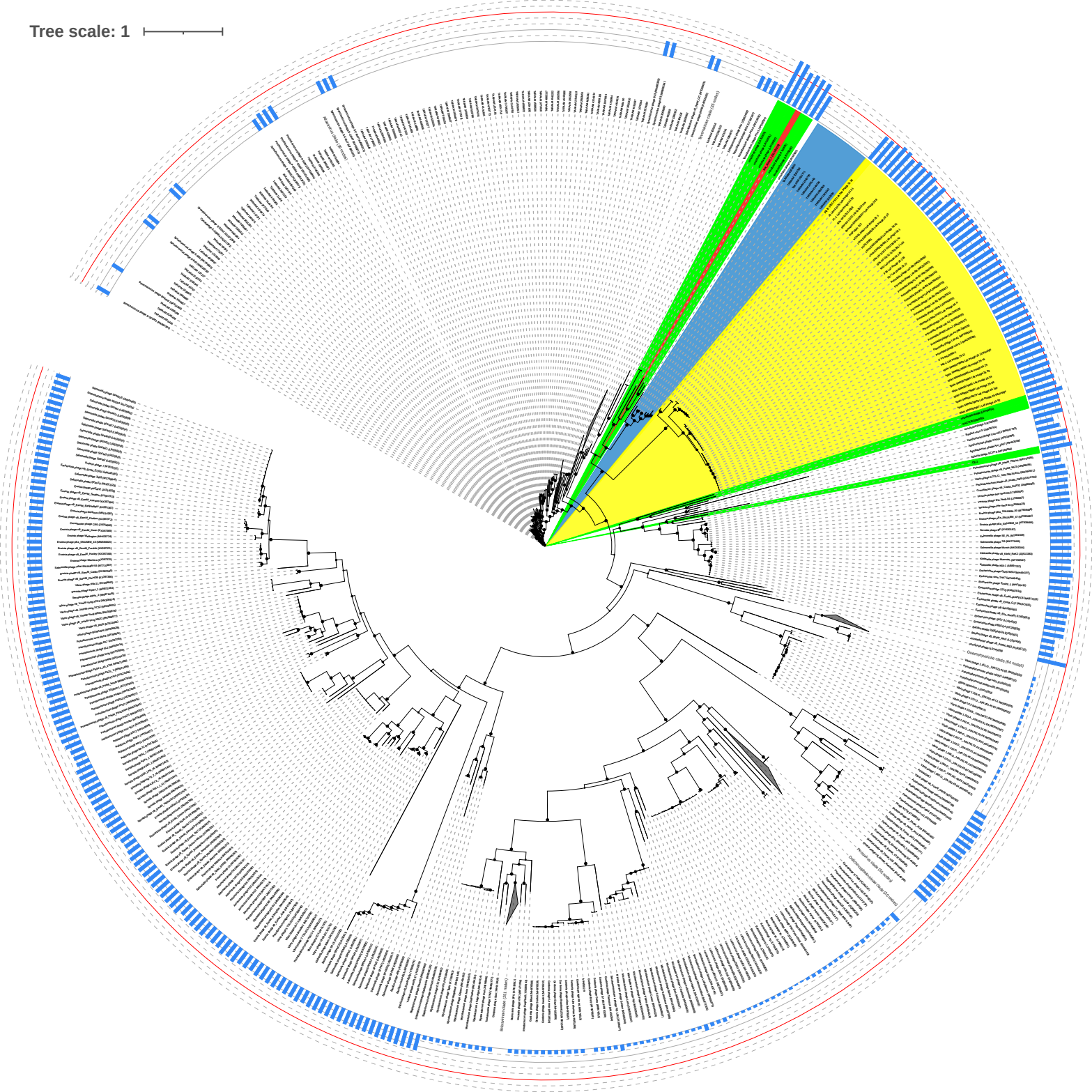

Supplement: Supplementary file 6 — Figure S3A [file 43705_2021_64_MOESM6_ESM.pdf]

Tree scale: 1

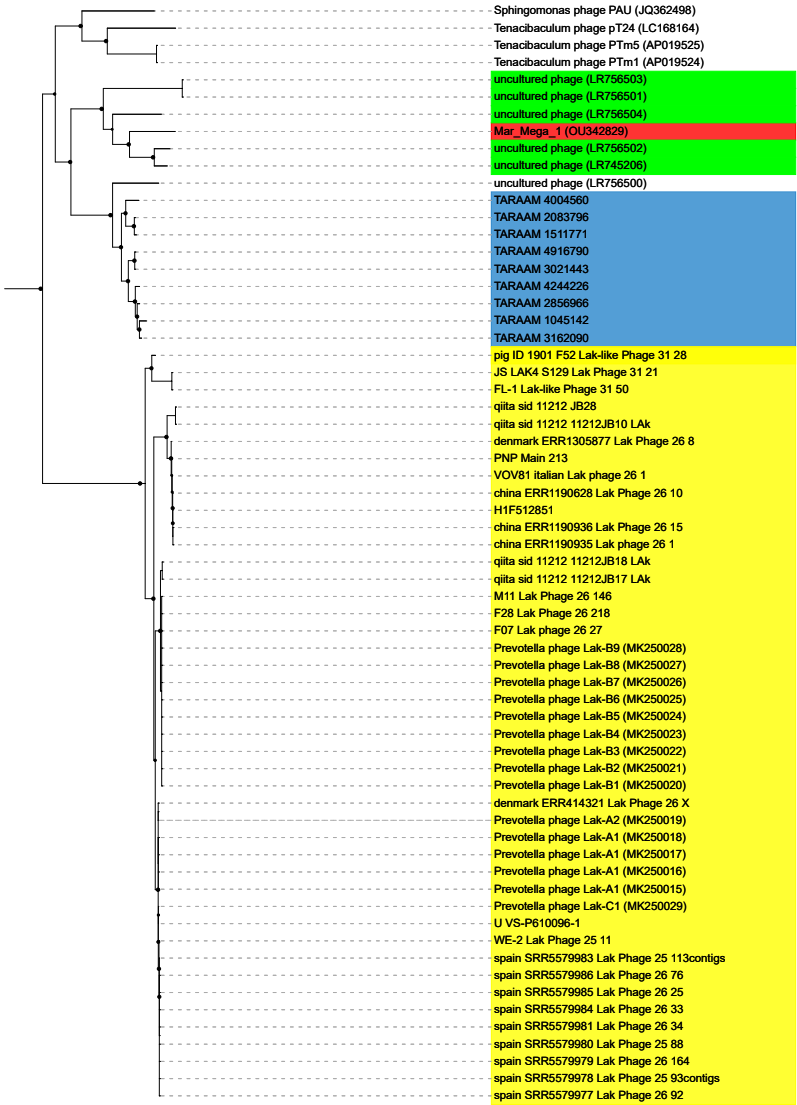

Supplement: Supplementary file 7 — Figure S3B [file 43705_2021_64_MOESM7_ESM.pdf]
